# Usability and acceptability of oral fluid hepatitis C self-testing among people who inject drugs in Coastal Kenya: a cross-sectional pilot study

**DOI:** 10.1186/s12879-022-07712-9

**Published:** 2022-09-15

**Authors:** Elena Ivanova Reipold, Emmanuel Fajardo, Emily Juma, David Bukusi, Elkin Bermudez Aza, Muhammad S. Jamil, Cheryl Case Johnson, Carey Farquhar, Philippa Easterbrook, Aliza Monroe-Wise

**Affiliations:** 1 FIND, Campus Biotech, Chemin des Mines 9, 1202 Geneva, Switzerland; 2 VCT and HIV Prevention Unit, Kenyatta National Hospital, Nairobi, Kenya; 3 Department of Global HIV, Hepatitis and STIs Programmes, World Health Organization, Geneva, Switzerland; 4 Departments of Global Health, Epidemiology and Medicine, University of Washington, Seattle, WA USA; 5 Department of Global Health, University of Washington, Seattle, WA USA

**Keywords:** HCV, PWID, Diagnosis, Self-testing, Oral fluid

## Abstract

**Background:**

People who inject drugs (PWID) are disproportionally affected by hepatitis C virus (HCV) infection and many remain undiagnosed. HCV self-testing (HCVST) may be an effective approach to increase testing uptake, but has rarely been used among PWID. We assessed the usability and acceptability of HCVST among PWID in Kenya.

**Methods:**

We conducted a cross-sectional study nested within a cohort study between August and December 2020 on Kenya’s North Coast region. Participants were handed a prototype oral fluid HCVST kit and asked to conduct the test relying on the instructions for use. Usability was assessed by documenting errors made and difficulties faced by participants. Acceptability was assessed using an interviewer-administered semi-structured questionnaire.

**Results:**

Among 150 participants, 19% were female and 65.3% had primary level education or lower. 71.3% made at least one error, 56.7% experienced some difficulty during at least one step, and the majority of participants (78%) required assistance during at least one step of the procedure. Most common errors occurred when placing the tube into the stand (18%), collecting the oral fluid sample (24%) and timing of reading results (53%). There was a strong association between presence of symptoms of opiate withdrawals and observed errors (94% vs 62%; p = 0.016) in a sub-group of 74 participants assessed. Inter-reader and inter-operator concordance were 97.7% (kappa: 0.92) and 99.2% (kappa: 0.95), respectively. Acceptability assessed by asking whether participants would choose to use HCVST prior to and after conducting HCVST was 98% and 95%, respectively.

**Conclusions:**

We found a high acceptability of oral fluid HCVST among PWID. User errors were common and were associated with the presence of withdrawal symptoms among users. Despite errors, most participants were able to obtain and interpret results correctly. These findings suggest that this group of users may benefit from greater messaging and education including options to receive direct assistance when self-testing for HCV.

**Supplementary Information:**

The online version contains supplementary material available at 10.1186/s12879-022-07712-9.

## Background

Hepatitis C virus (HCV) is one of the leading causes of viral hepatitis, cirrhosis, and liver cancer worldwide, with an estimated 71 million people living with the virus and nearly 400,000 annual HCV-related deaths [1]. The global HCV burden is disproportionately distributed in low- and middle-income countries [2], where 80% of the world’s HCV-infected population live and where significant barriers to accessing testing, care and treatment may impede advances in controlling the burden of disease [3]. As of 2019, the World Health Organization (WHO) estimates that globally less than one quarter of all persons with HCV infection had been diagnosed and nearly 40% of diagnosed persons remain untreated [4].

In 2016, the WHO launched a Global Health Sector Strategy on Viral Hepatitis, with ambitious goals to eliminate HCV as a public health threat by 2030, defined as an 80% reduction in new HCV infections and a 65% reduction in HCV-related mortality by 2030 [5]. To achieve these impact targets, modelling indicated that this would require 90% testing coverage of eligible populations, and 80% treatment coverage of those HCV infected. However, global progress toward these goals was already falling short by 2019 with only 21% testing coverage and 62% treatment coverage [6].

With the availability of highly effective, low-cost generic direct acting antiviral therapy offering cure rates of over 90% [7, 8], the global HCV response has turned towards expansion of testing and treatment programs. In 2018 the WHO recommended a “treat all” approach for all people aged > 12 years infected with HCV regardless of disease stage or population [9]. The 2017 WHO testing guidelines for hepatitis B and C also recommended routine focused HCV testing for the most affected populations that include people who inject drugs (PWID) and men who have sex with men (MSM), people in prisons, as well as access to general population screening for those settings and countries with a general population prevalence ≥ 2% [10, 11] using a single rapid diagnostic test (RDT) or laboratory-based assay. However, access to HCV testing services remains a barrier to achieving HCV elimination goals, particularly among high-risk populations [3], and nearly 80% of infected individuals worldwide remain undiagnosed [4, 12].

PWID are among the highest risk groups for HCV worldwide [13]. In Kenya, PWID constitute the highest risk group with a prevalence estimated between 13 and 40% [14–16] compared with < 1 to 4% in the general population [17, 18]. PWID also experience barriers to accessing care that result in low rates of testing, engagement in care, and completion of treatment courses [19–21]. Of Kenya’s estimated 115,000 HCV-infected individuals, fewer than 25% have been diagnosed [22]. Expansion of HCV testing and treatment services that are accessible to vulnerable populations is vital to make progress toward the WHO’s ambitious testing and treatment goals. A recent systematic review showed that full decentralisation and integration of hepatitis C testing and treatment at harm reduction sites—a “one-stop shop” resulted in increased uptake of testing, linkage and treatment among PWID [23].

Self-testing (ST) is a testing approach in which people, at a time and place of their choosing, can collect their own specimen, perform a rapid test, and then read and interpret the results. While self-testing has been used extensively for pregnancy and certain chronic conditions such as blood glucose monitoring in diabetes for decades, the use has increased in the past decade for infectious diseases such as HIV and malaria. WHO recommends HIVST as safe, accurate and effective at increasing the uptake and frequency of testing among high risk populations such as MSM and female sex workers [24–27], while achieving comparable positivity and linkage rates to standard HIV testing [26, 28, 29].

Kenya was an early adopter of HIVST, undertaking studies among healthcare workers as early as 2006 and including HIVST in the national HIV testing guidelines in 2015 [30]. As a result, there has been extensive implementation of HIVST and the practice is now widely acceptable in the general population, key populations, and among individuals taking pre-exposure prophylaxis [31, 32]. However, acceptability and other features of HIVST have not been widely studied among PWID in Kenya or in other countries despite their high risk. Self-testing for HCV antibodies is a novel approach, with data limited to a few small pilot studies in the general population using experimental testing products that showed high agreement between results of self-testing and healthcare provider-delivered testing [33, 34]. Acceptability of HCVST was found to be high in one small study of PWID in London, but concerns were highlighted about access to facility based confirmatory viral load testing and care [35]. To better understand usability and acceptability of HCVST among key populations, the WHO has partnered with the Foundation for Innovative New Diagnostics (FIND) to coordinate a series of pilot studies of acceptability and usability of HCVST in different populations in five countries—Egypt (general population in semi-rural community in high burden country) [36], China (MSM), Vietnam (PWID and MSM) [37], Georgia (PWID and MSM) and Kenya (PWID and their partners). Based on preliminary results of these pilots and other data, WHO published HCVST recommendations and guidance in 2021 [38]. Here we describe results from the pilot study conducted among PWID living in the coastal region of Kenya, the objectives of which were to understand the acceptability and usability of HCVST among PWID in Kenya.

## Methods

### Parent study

We conducted a cross-sectional observational study nested within a large ongoing longitudinal parent study among PWID and their partners in Kenya, the protocol for which was recently published [39]. The parent study, entitled “SHARP,” enrolls HIV-positive people who inject drugs as “index” participants and then employs assisted partner services to identify, test, and link to care the index’s sexual and injecting partners. Inclusion criteria for SHARP differed based on whether individuals were enrolling as index participants or as partners. Index participants in SHARP were HIV-positive PWID 18 years of age or older who had injected drugs at least once in the past year. Inclusion criteria for partners in SHARP were individuals 18 years of age or older who had engaged in sexual intercourse with index cases at least once in the previous 3 years, or who had injected drugs in the presence of index cases at least once in the previous 3 years. All index participants and partners are tested for HCV at baseline enrollment using RDTs for HCV antibodies.

### Nested sub-study

#### Study design

The nested study we describe herein was a cross-sectional sub-study that recruited from any participant newly enrolling as an index or as a partner participant in SHARP.

#### Setting & population

Participant recruitment and data collection took place at multiple settings located in Mombasa and Kilifi counties. Study procedures took place in partnership with three organizations that operate drop-in centers and provide harm reduction (needle and syringe programmes), social services, and HIV testing and care services to PWID on Kenya’s coast: ReachOut in the city of Mombasa, Muslim Education and Welfare Association in Mtwapa and Kilifi towns, and Omari Project in Malindi town. Outreach services are conducted by community-embedded peer educators who are former drug users.

Participants in this study were individuals aged 18 years or older who were either HIV-positive people who had injected drugs in the previous year, or were individuals aged 18 years or older who had had sexual intercourse or had injected with an HIV-positive person who injects drugs in the previous 3 years... The target sample size for the nested study was 150 participants, calculated to detect at least 50% HCVST acceptance rate with a 10% margin of error.

#### Sampling & recruitment

Individuals considered for participation this study were identified during screening and enrollment for SHARP, the parent study. Any individual newly enrolling in SHARP was considered for enrollment in the HCVST sub-study on a first-come first-served basis between August and November, 2020. Potential participants were informed of a secondary study to evaluate self-testing for HCV by study staff, and those participants who were interested in enrolling in the sub-study underwent a secondary screening process immediately following screening for the parent study.

#### Inclusion & exclusion criteria

In addition to meeting the eligibility criteria for the parent study, participants in this study were excluded if they had previously ever tested positive for HCV antibody, or if they had tested negative for HCV antibody within the past year. They were also ineligible if they had ever completed a self-test for HIV or HCV. The purpose of these criteria was to exclude any potential participants who might be biased in their interpretation of the HCVST results or acceptance of HCVST, either due to previous testing or due to previous experiences with self-testing. While participants in the sub-study were required to have also enrolled in the parent study, participation in the sub-study was not a requirement for enrollment in the parent study.

#### Procedures

Following screening, eligible participants provided written informed consent to participate in both the parent study and the sub-study. Participants received 400 Kenya Shillings (KSh, about $4) as reimbursement for time and travel for enrollment into the parent study, and an additional KSh 200 (about $2) for time spent participating in the HCVST sub-study. Enrolled participants completed a baseline questionnaire that covered socio-demographic characteristics, sexual and injecting risk behaviors, and previous experiences with HIV and HCV testing and care. Sexual and injecting risk behavior questions included questions about ever engaging in unprotected anal intercourse, any injecting unprescribed drugs, any needle sharing, surgical or dental procedures, tattoos, and sharing shaving equipment.

Following the baseline questionnaire, participants performed the HCV self-test while being observed by study staff in private rooms within the drop-in centers. We used prototype self-testing kits that included a professional use OraQuick® HCV Rapid Antibody Test (OraSure, Inc., US), a plastic stand and instructions for use (IFU) adapted for self-testing by the test manufacturer. Each participant was given the kit and told to follow the procedures written on the IFU. Instructions were mostly pictorial, but included key words or phrases written in the local language Swahili (Additional file 1: Fig. S1). Study staff noted both errors and difficulties with self-testing steps according to a standardized checklist, while observing the participant complete the self-testing procedure. The testing checklist included questions on pre-testing steps (opening the pouch, removing contents, reading instructions, removing the test tube from its package, removing the cap from the test tube, placing the test tube in the stand, and removing the test device), testing steps (correct handling of the test device without touching the flat pad, collection of oral fluid specimen, placing the test device in the test tube and keeping time correctly) and test interpretation steps (interpreting the results correctly).

Participants were encouraged to perform the self-test on their own without assistance. If participants had trouble with steps in the self-testing procedure and requested assistance, study staff provided assistance only after the participant had made multiple attempts at completing the step, usually after 15 min. Staff also noted which steps participants required assistance with. Self-testing results were first read and interpreted by the participant, and then the same results were read and interpreted by the study staff member, and both interpretations were recorded in a test result form.

Participants who self-tested also completed a questionnaire to ascertain opinions and experiences of the self-testing procedure. Questions included the participants’ rating of the test’s ease of use, their willingness to use the test again or to recommend it to family or friends, preferred testing modalities (oral fluid or blood-based), and their understanding of actions they need to take if they tested positive.

Finally, all participants were tested by a trained healthcare worker with the OraQuick® HCV Rapid Antibody Test for professional use. Results from this second test were read and interpreted by study staff, communicated to the participants, and recorded in the test result form. Post-test counselling was provided to all participants, and those found to be positive were linked to confirmatory RNA testing and HCV treatment as part of routine SHARP procedures.

#### Data management & analysis

Data were all collected electronically and kept in two separate databases. The baseline questionnaire was administered by trained study staff using the Open Data Kit platform on handheld tablets, and these data were stored in the SHARP database on servers within Kenya’s Ministry of Health (MoH). All other data including the self-testing checklist and post-testing questionnaire were collected through OpenClinica, an online data collection system, and stored in the OpenClinica database. Variables of interest from the baseline questionnaire were extracted from the MoH server and merged with the OpenClinica dataset. Data cleaning and analysis were performed using STATA v.14 (Texas, USA).

Usability was assessed through a combination of percentage of observed errors and difficulties and calculation of inter-reader and inter-operator concordance. Baseline characteristics and errors, difficulties, and assistance required in self-testing were compiled and analyzed using simple frequencies and percentages. Errors, difficulties and assistance variables were also compounded into aggregate variables describing whether any errors (problems or omissions noted with any step on the checklist), difficulties (troubles with steps that did not cause errors) or assistance (help provided with any step and noted in the checklist) occurred. Inter-reader concordance was calculated as the percentage agreement between the participant’s and the staff’s interpretation of the participant’s self-test result, including invalid results but excluding participants who received assistance reading the results. Inter-operator concordance was calculated as the percentage agreement between the participant’s interpretation of the self-test result and the staff’s interpretation of the professional use test result, excluding invalid results and excluding participants who received assistance reading the results. Cohen’s Kappa coefficient was calculated for both. Acceptability was assessed through percentages of self-reported opinions and experiences of the self-testing procedures.

About half-way through enrollment in the study, study staff noted that individuals who were experiencing symptoms or showing signs of opioid withdrawal appeared to have greater difficulty with self-testing procedures. After this observation, the baseline questionnaire was modified to include a question on whether each person was experiencing symptoms or showing signs of withdrawal. We then conducted Pearson chi-square and Fisher’s exact tests to assess whether experiencing withdrawal symptoms was associated with making errors, experiencing difficulties, or requiring assistance during the testing process, respectively. Fisher’s exact test was used when the expected frequency was less than 5 in any given category, whereas Pearson chi-square tests were used for the remaining comparisons.

## Results

### Participant characteristics

A total of 186 individuals were screened for participation. Of these, 150 were eligible, all of whom enrolled in the study. Participants had a median age of 35 years, were predominantly male (79.3%), and 65.3% had attained only primary level education or lower (Table 1). Nearly all (96.6%) were PWID; 87.3% reported having injected drugs in the past month (currently injecting) and 9.3% reported injecting drugs in their lifetime but not in the past month. Other risk behaviors were common, with 23.3% reporting unprotected anal intercourse and 40% sharing of needles. Awareness of self-testing was high, and 80% of participants reported that they were aware that some tests could be performed at home.

**Table 1:** Baseline demographic characteristics

	N = 150	%
Age, years [median (range)]	35	(21–59)
Sex		
Female	29	19.3
Male	119	79.3
Missing	2	1.3
Education level		
Primary school or less	98	65.3
Secondary school	41	27.3
College or higher	11	7.3
Employment status		
Formally employed	54	36.0
Informally employed	82	54.7
Unemployed	11	7.3
Not available	3	2.0
Marital status		
Married	61	40.7
Unmarried	57	38.0
Divorced or widowed	30	20.0
Not available	2	1.3
Injecting status		
Never injected	5	3.3
Formerly injected	14	9.3
Currently injecting	131	87.3
Self-reported exposures (ever) to HCV risk factors		
Unprotected anal intercourse	35	23.3
Injecting unprescribed drugs	112	74.7
Sharing needles	60	40.0
Surgical procedures	30	20.0
Dental procedures	42	28.0
Sharing shaving tools or toothbrushes	57	38.0
Tattoo	30	20.0
None reported	4	2.7
Frequency of routine health check per year		
More than once per year	87	58.0
Once per year	26	17.3
Rarely	9	6.0
Never	28	18.7
HIV status		
Positive	47	31.3
Negative	99	66.0
Unknown	4	2.7
Awareness about self-testing		
Aware that tests can be performed at home	120	80.0

Withdrawal question	N = 74	%
Currently experiencing withdrawal symptoms	16	21.6

Since the question about withdrawal symptoms was added half-way through the study, only about half of participants (74 individuals, 49.3% of all participants) provided information on withdrawal symptoms. Of these, 21.6% reported experiencing withdrawals at the time of data collection.

### Usability

Overall, 71.3% of participants made at least one error, 56.7% experienced difficulties during at least one step, and the majority of participants (78%) required assistance during at least one step of the procedure (Table 2). Only 14.7% were able to perform the procedure without any errors, difficulties, or assistance.

**Table 2:** Observer assessment of errors, difficulties and steps requiring assistance

Observation	N = 150	%
Observed errors at each step		
Pre-testing		
1. Opening the package	0	0
2. Reading/using the instructions for use	2	1.3
3. Removing the test tube from the test pack	0	0
4. Removing the cap from the test tube	4	2.7
5. Placing the tube into the stand	27	18
6. Removing the test device from the test pack	0	0
Conduct of the test		
7. Touching the flat pad	9	6
8. Incorrect manipulation to collect oral fluid	36	24
9. Wrong placing of the test device in the test tube	9	6
10. Test device came out of the tube while testing	0	0
11. Spilling fluid or pouring fluid out of the tube	17	11.3
12. Improper time keeping	80	53.3
Observed difficulties with testing steps		
1. Opening the package	27	18
6. Sliding the tube into the stand	72	48
Assistance provided		
1. Opening the package	21	14
2. Opening and removing the cap from the tube	6	4
3. Placing the tube into the stand	69	46
4. Placing the test device into the tube	11	7.3
5. Keeping time	56	37.3
6. Reading the results	17	11.3
Errors observed for at least one step	107	71.3
Experienced difficulties for at least one step	85	56.7
Assistance provided for at least one step	117	78
Completed all testing steps correctly without any assistance and interpreted the test results correctly	22	14.7

Four steps in the self-testing process accounted for the majority of errors: placing the tube into the stand (18%), collecting the sample incorrectly (24%), spilling or pouring fluid from the tube (11%) and timing the test incorrectly (53.3%). Of those who made errors placing the tube into the stand, 41% requested assistance, 27% either held the test tube or placed it on the table, and 11% either poured the fluid into the stand or were assisted as they attempted to pour fluid into the stand. Of those who made errors collecting the sample, the majority (69%) swabbed the upper and lower gums more than once, and a small number (8%) swabbed upper or lower gums but not both or placed the flat pad on the gums but did not swipe (8%). Of those who made errors keeping time, the majority (78%) read the results early, many of whom were noted to have seen that the results were “ready” before the appropriate time. Others were noted to have not kept track of time at all. Of the 46 participants who read the results at the incorrect time, 65% had a time keeping device; while of the 104 participants who read the results at the correct time, 27% did not have a time keeping device.

Observed difficulties that did not result in errors occurred during package opening and during the process of sliding the tube into the stand. Twenty-seven participants (18%) had difficulty opening the package, while 48% had difficulty sliding the tube into the stand.

Many participants required assistance in completing certain self-testing steps: 14% in opening the package, 46% in placing the tube into the stand, 37.3% in keeping time, and 11.3% in reading the results.

Among the subset of participants who provided data about their experience of withdrawals (Table 3), withdrawal symptoms were significantly associated with conducting an error at any step of the procedure (Fisher’s exact test p-value 0.016).

**Table 3:** Association between withdrawals and errors

	Withdrawals (N = 16)	No withdrawals (N = 58)	P value^a^
Any errors			
Yes	15 (93.8)	36 (62.1)	0.016
No	1 (6.2)	22 (37.9)	
Any difficulties			
Yes	12 (75.0)	34 (58.6)	0.262
No	4 (25.0)	24 (41.4)	
Required assistance			
Yes	15 (93.8)	42 (72.4)	0.097
No	1 (6.3)	16 (27.6)	
Total			

^a^Fisher’s exact test

**Fig. 1:**
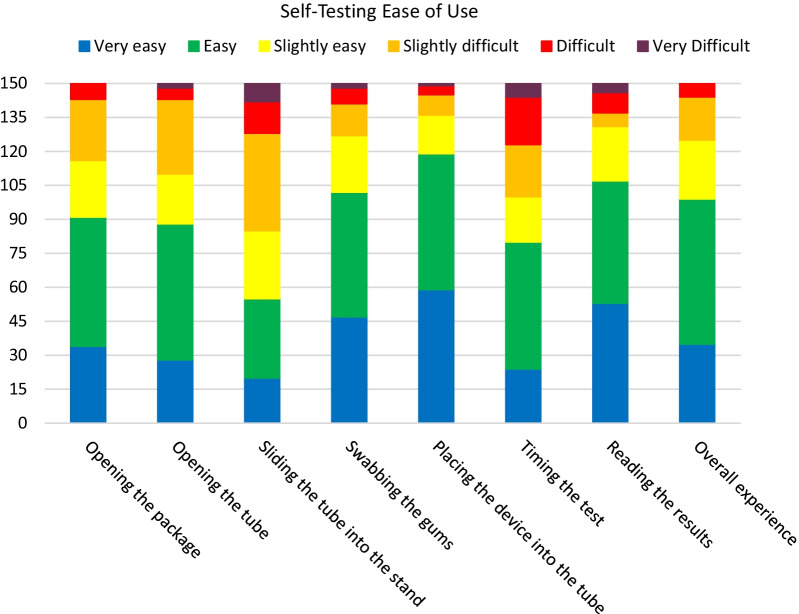
Ease of use ratings

Participants reported that the self-testing steps were easy to perform, on the whole (Fig. 1). Steps that were associated with the highest ease of use ratings were swabbing the gums (68% reporting very easy or easy), placing the device into the tube (79% reporting very easy or easy), and reading the results (71% reporting very easy or easy). Conversely, steps that were reported to be more difficult included sliding the tube into the stand (15% reporting very difficult or difficult) and timing the test (18% reporting very difficult or difficult). Overall testing experience was largely reported to be easy, with 66% reporting that the testing procedure was very easy or easy.

### Concordance of results

Despite the challenges that participants experienced during the self-testing procedure, concordance was high, both between readers and between operators (Table 4). Of the 132 participants who completed the self-testing procedure without assistance in reading the results, 129 interpreted the results in concordance with the trained staff’s interpretation, including 8 who correctly interpreted the result as being invalid (inter-reader concordance of 97.7%, Cohen’s Kappa of 0.92). Inter-operator concordance, the agreement between the participant’s read of the self-test and the staff member’s read of the professional test, was 99.2% with a Cohen’s Kappa of 0.95, indicating moderate to substantial agreement. We excluded data pairs containing self-testing results interpreted by participants as invalid (9 data pairs) and those where participants were unsure of the self-test results (1 data pair) from inter-operator concordance calculations. The most common scenarios for disagreement were among tests interpreted as invalid during the self-testing process (n = 9), of which 2 were positive and 7 were negative during professional use testing.

**Table 4:** Assessment of inter-reader (left panel) and inter-operator (right panel) concordance

Participant Assessment	Total	Re-reading of self-test by trained staff			Re-tested with professional kit by trained staff	
		Positive	Negative	Invalid	Positive	Negative
Positive	12	11	1	0	11	1
Negative	110	0	110	0	0	110
Invalid	9	0	1	8		
Unsure	1	0	0	1		
Total		11	112	9	11	111
Concordance (%)		97.7%			99.2%	
Cohen’s Kappa		0.92			0.95	

### Acceptability

Self-testing for HCV was acceptable to the large majority of participants. Before undergoing the self-testing procedure, nearly all participants stated they would self-test for HCV if it was available (98%, Table 5). After self-testing, over 95% of participants said they would use the kit again and would recommend and/or deliver HCVST to friends and family. Six out of the seven participants who said that they were either not sure or would not use the self-test again had faced difficulties during the self-testing process and required assistance. Most (80%) also said they would prefer testing alone at home, and 134 (89.3%) said they would prefer oral fluid testing. Nearly all participants (99.3%) stated that they would contact a healthcare facility if they had a reactive self-test result.

**Table 5:** Participant views and preferences regarding HCV self-testing

	N = 150	%
Before self-testing		
Eligible subjects who agreed to participate and perform HCV self-testing	150	100
Participants who would use HCV self-testing if it was available	147	98
Post-testing acceptability		
Would use HCV self-test again	143	95.3
Would recommend the HCV self-test to family members/friends	145	96.7
Take the test to family members/friends	144	96.0
Preferences on HCV self-testing		
Preferred approach to test for HCV in the future		
By myself at home	120	80
By myself at a health center	27	18
In a community center by a healthcare worker	25	16.7
In a screening campaign	2	1.3
Preferred sample type		
Prefer oral fluid-based test	134	89.3
Prefer blood-based test	14	9.3
No preference	2	1.3
Steps to take if results of self-test positive		
Contact healthcare facility	149	99.3
Contact pharmacy	2	1.3
Do a confirmatory test	2	1.3
Seek advice from a family member or friend	3	2
Seek advice from an NGO community representative	6	4
Do not know	0	0
Knowledge about HCV treatment		
Know that HCV can be cured	54	36
Know that there is a treatment but not sure about cure	23	15.3
Not sure if there is treatment	21	14
There is no treatment or cure	4	2.7
No idea if there is treatment or cure	48	32

Knowledge about HCV treatment was low. Only 36% of all participants knew that HCV can be cured, and 32% said they had no idea if there was treatment or a cure.

## Discussion

Increasing access to HCV testing among high-risk groups in low- and middle-income countries is essential to achieve WHO viral hepatitis elimination goals by 2030. Recently published reports showed high usability and acceptability of HCV self-testing in the general population in Egypt, South Africa and Rwanda [36, 40, 41] as well as in high-risk populations in Vietnam [37]. We conducted the first study to assess the usability and acceptability of self-testing for HCV among PWID in Kenya. We found that critical errors in performing the self-testing procedure were uncommon and most participants were able to obtain and interpret results accurately. Although many participants required assistance, it is unclear whether the assistance was necessary for the accuracy of self-testing results. Increased demand for assistance in self-testing process was observed in a similar study conducted among PWID in Vietnam [37]. Participants who were actively withdrawing from opioids were more likely to encounter errors and difficulties in this population. Acceptability was very high, with almost all participants agreeing that they would use the test again in the future and share the test with family or friends.

Many of the most common issues participants encountered in conducting the test were addressable through modifications in the packaging or instructions. Many participants were confused by the step of putting the test tube into the stand, resulting in some errors and some requests for assistance. Similarly, many participants did not perform the self-swabbing technique correctly, with many swabbing more times than required, and many read the results at the incorrect time. Previous studies on usability of oral fluid based HCV self-tests also reported difficulties with these two steps [36, 40]. For the majority, these problems did not result in invalid test results; nonetheless, this confusion could be addressed by modifying the test tube and/or the stand, clarifying written or pictorial instructions, or providing additional training or materials before self-testing, including videos. Instructions should make it clear that a time keeping device is necessary, as this was a frequently encountered barrier to completing the test. Some encountered difficulties opening the package, which could be modified to improve ease of use.

In our population, many individuals were experiencing symptoms of withdrawal from opioids while undertaking the self-testing procedure, and withdrawals were significantly associated with encountering difficulties or making errors in the testing process. Consideration should be made for recommendations about when self-testing should be performed in this population, with messaging around avoiding self-testing while experiencing withdrawals, and further studies are warranted to assess whether this messaging is effective at reducing errors and difficulties during self-testing in this population.

Despite the number of individuals who encountered difficulties, made mistakes, or needed assistance while conducting the test, concordance of results was high. There was one individual whose read of the self-test was positive, but whose read of both the self-test and the professional use test by a professional was negative. Although this falsely positive test interpretation is unfavorable, it was extremely uncommon (0.6%) and is a more favorable outcome than the converse, in which a participant may read a positive test result as negative. Self-testing resulted in 9 invalid test results as determined by the interpretation of the healthcare worker. Of these, 4 (44%) poured fluid into the stand and then either placed the test device into the stand or onto the table, and 6 (67%) interpreted the results of the test before 20 min had passed.

Finally, knowledge about HCV testing and treatment is poor in this population, mirroring previous studies in other settings [20, 42]. Conversely, both awareness of self-testing for other infections and acceptability of HCVST were high, indicating an opportunity for the use of this testing modality to improve awareness of HCV among PWID. Kenya has had an existing government-led HIVST program since 2017 [43], which could be expanded to include HCVST, particularly for key populations. Leveraging existing HIVST systems and infrastructure would allow for improvements in knowledge and awareness of HCV testing among high-risk populations. Previous studies have shown that improving knowledge of HCV can increase HCV treatment willingness [42], which may be vital to scaling up treatment efforts. Concerted efforts should be made to expand awareness and knowledge of HCV testing and treatment availability, particularly among PWID.

Several limitations were present in our study. Our sample size was small and only included PWID and their partners living on Kenya’s coast, and therefore may not be generalizable to other populations. However, PWID are both understudied and vitally important to HCV control worldwide and our results may help inform policies for this population in other settings. Study participants were recruited from the population already engaged in harm reduction services and thus may not represent the PWID population in Kenya. The presence of an observer in the room during the self-testing process may have resulted in an abnormally high rate of requests for assistance, biasing the results. Further studies are necessary to assess whether the reliability of self-testing in this population is similar in differing testing environments. Our study was also limited to oral fluid testing and was not able to assess acceptability of other testing modalities, including fingerstick blood testing.

Our study demonstrated high inter-reader and inter-operator reliability of HCVST among PWID in Kenya, who were largely able to perform ST correctly and produce accurate results despite making minor errors. Improvements in the testing packaging and instruction booklet or alternative modalities of self-testing delivery, including staff or peer educator demonstrations or videos prior to testing, should be considered. Messaging around self-testing for PWID should acknowledge the user’s withdrawal symptoms, with a suggestion that the test should not be performed while people are experiencing withdrawals. Finally, efforts to improve knowledge of HCV among PWID should be made at the policy-level, as this may augment campaigns to find and treat individuals in this population. Integrating HCVST within Kenya’s HIVST program would allow for leveraging of distribution channels, training mechanisms, and help to build awareness.

## Supplementary Information

**Additional file 1: Figure S1.** Instructions for Use of OraQuick® HCV Rapid Antibody Self Test (OraSure, Inc., US).

## Data Availability

Datasets used and/or analyzed are available from the corresponding author on reasonable request.

## Abbreviations

FIND: Foundation for Innovative New Diagnostics
HCV: Hepatitis C virus
HCVST: Hepatitis C virus self-testing
HIV: Human immunodeficiency virus
HIVST: Human immunodeficiency virus self-testing
MOH: Ministry of Health
MSM: Men who have sex with men
PWID: People who inject drugs
RDT: Rapid diagnostic test
ST: Self-testing
WHO: World Health Organization
