# Supplementary material for: Usability and acceptability of oral fluid hepatitis C self-testing among people who inject drugs in Coastal Kenya: a cross-sectional pilot study
Source: BMC Infect Dis. 2022 Sep 15;22:738. doi: 10.1186/s12879-022-07712-9 (PMC9479404; doi:10.1186/s12879-022-07712-9)
Supplement: Supplementary file 1 — Additional file 1: Figure S1. Instructions for Use of OraQuick® HCV Rapid Antibody Self Test (OraSure, Inc., US). [file 12879_2022_7712_MOESM1_ESM.pdf]

You must follow the test directions carefully to get an accurate result. Do not eat or drink for at least 15 minutes before you start the test or use mouth cleaning products 30 minutes before you start the test.

WARNING: if you are on HCV treatment (ARVs) you may get a false result.

OraQuick® Rapid Antibody Test HCV

OW TO USE THE OraQuick® HCV SELF-TEST KIT

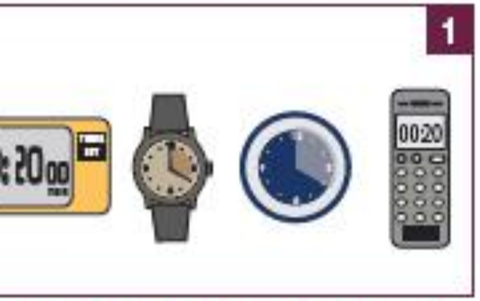

WILL NEED A WAY TO TIME THE TEST

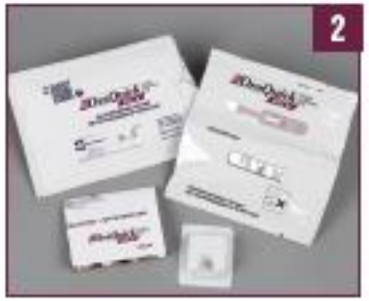

Pouch contains: **test kit, test stand and instructions.**

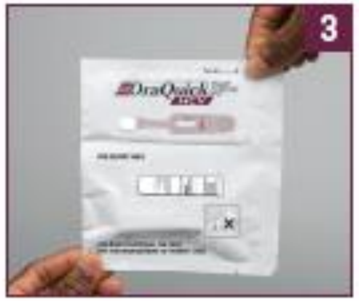

Your test kit contains two pouches.

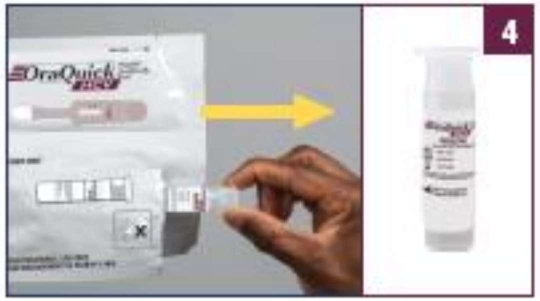

Tear open the pouch containing the **tube.**

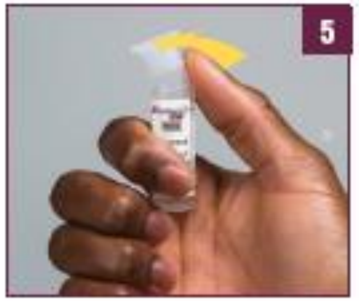

Remove the cap.

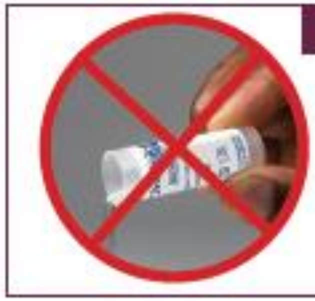

DO NOT pour out the **liquid.**

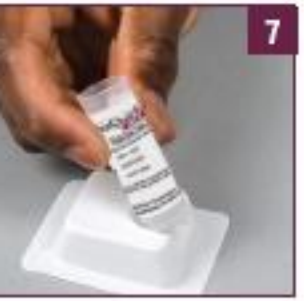

the tube into the **stand.**

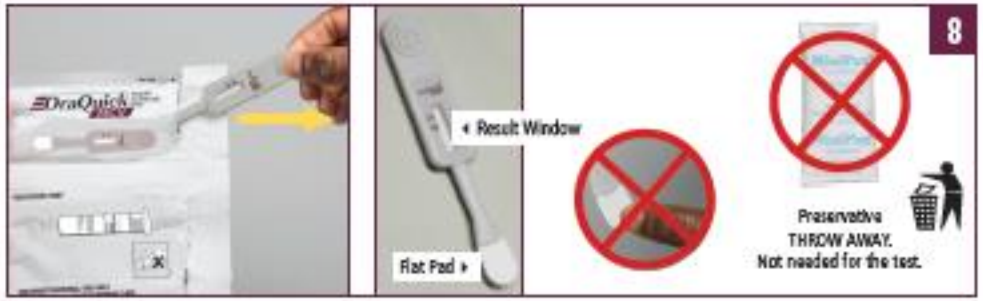

Tear open pouch containing the **test device** and remove. **DO NOT** touch the flat pad with your fingers.

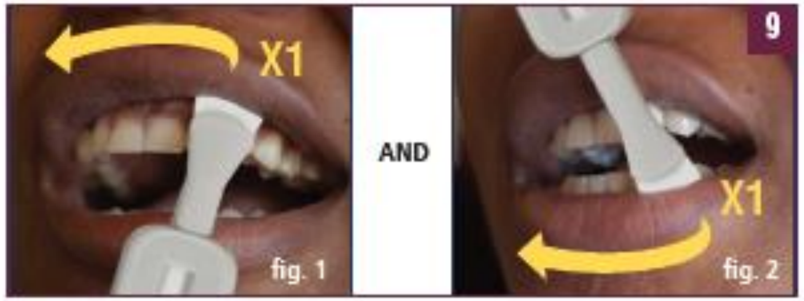

Press the **Flat Pad** firmly against your gum and swab it along your **upper gum once** (fig. 1) and **your lower gum once** (fig. 2).

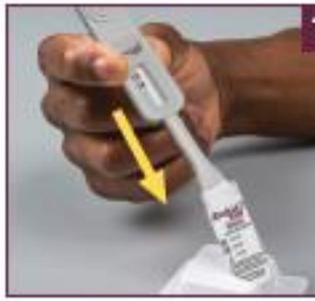

Slide the tube into the **stand.**

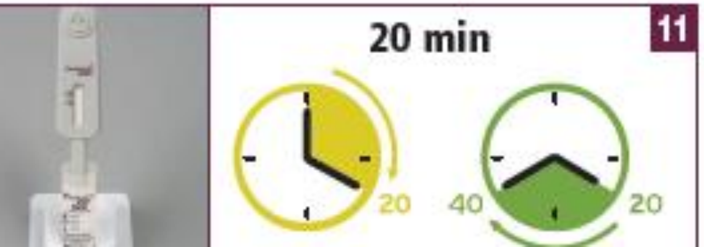

LEAVE IT THERE for 20 minutes before reading the results.
DO NOT read the result after 40 minutes.
Test again in 6 months.

DRAFT
02/21/17

FOR INVESTIGATIONAL USE ONLY
NOT FOR PATIENT CARE

INTERPRETING RESULTS

HCV POSITIVE RESULT

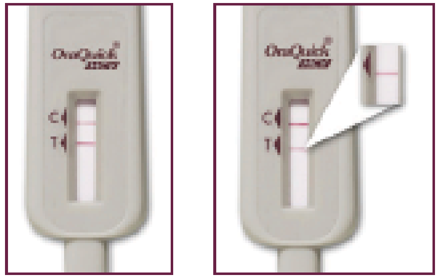

TWO LINES, even if the line is faint, means you may be HCV POSITIVE and you need to seek additional testing.

As soon as possible . . .

Visit your nearest
HCV Testing Center or Health Facility

HCV NEGATIVE RESULT

IF READ BEFORE 20 MINUTES, RESULT MAY NOT BE CORRECT

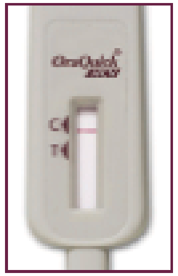

ONE LINE next to the "C" and NO line next to the "T", your result is HCV NEGATIVE.

Seek regular testing. If you may have been exposed to HCV test again in 3 months.

INVALID RESULT

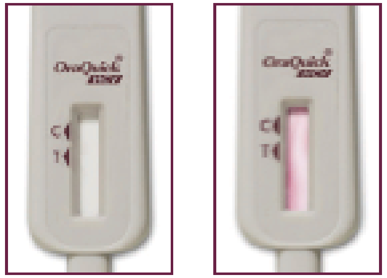

No line next to the "C" (even when there is a line next to the "T"), or a red background makes it impossible to read the test, the test is not working and should be repeated.

You will need to obtain another test.

The test did not work properly.

Visit your nearest HCV Testing Centre or Health Facility to test again.

NOT SURE OF RESULT

You do not know your result or you are unsure of your result.

Visit your nearest HCV Testing Centre or Health Facility to test again.

DISPOSE

Remove the test stick, put the cap on the test tube and throw away all contents in the normal trash.

Manufactured in Thailand for:
OraSure Technologies, Inc.
220 East First Street
Bethlehem, PA 18015
01+610-882-1820 • www.OraSure.com
